# Fracture fixation versus revision arthroplasty in Vancouver type B2 and B3 periprosthetic femoral fractures: a systematic review

**DOI:** 10.1007/s00402-020-03332-7

**Published:** 2020-02-21

**Authors:** Karl Stoffel, Michael Blauth, Alexander Joeris, Andrea Blumenthal, Elke Rometsch

**Affiliations:** 1 Department of Orthopedics and Traumatology, Kantonsspital Baselland, Bruderholzspital, 4101 Binningen, Switzerland; 2 University of Basel, 4000 Basel, Switzerland; 3 Department of Trauma Surgery and Sports Medicine, Medical University of Innsbruck, Anichstrasse 35, 6020 Innsbruck, Austria; 4 AO Clinical Investigation and Documentation, AO Foundation, Stettbachstrasse 6, 8600 Duebendorf, Switzerland

**Keywords:** Periprosthetic fracture, Femoral fracture, Hip arthroplasty, Review, Aged patients, Vancouver classification

## Abstract

**Introduction:**

Hip arthroplasty (HA) is commonly performed to treat various hip pathologies. Its volume is expected to rise further due to the increasing age of the population. Complication rates are low; however, periprosthetic femoral fractures (PFF) are a rare, albeit serious, complication with substantial economic impact. While current guidelines propose revision with long-stemmed prostheses for all Vancouver B2 and B3 PFF, some recent research papers suggest that open reduction with internal fixation (ORIF) could lead to an equivalent outcome. Our aim was to summarize the evidence, elucidating under which circumstances ORIF leads to a favorable outcome after B2 and B3 PFF compared with revision surgery.

**Materials and methods:**

A systematic literature search was performed to identify studies on patients treated with ORIF and with stem revision after B2 and/or B3 fractures. Extracted information included initial pathology, stem fixation mechanism, bone quality and stem stability at the time of PFF, clinical outcomes, and mortality. Results of individual studies were summarized in a table in lieu of a quantitative data synthesis due to a lack of standardized information.

**Results:**

We identified 14 original research articles including both patients treated with ORIF and with stem revision after B2 and/or B3 PFF. Five studies included statistical comparisons, all were in favor of ORIF or indeterminate. The common lack of rigorous statistical analyses and significant methodological weaknesses made identification of outcome predictors impossible.

**Conclusion:**

The choice of treatment modality for PFF depends on fracture, implant, and bone characteristics. Recent data show that successful outcome can be achieved without revising loose stems. ORIF may be a viable option if bone stock is adequate around uncemented or tapered polished stems with an intact cement mantle and the fracture geometry allows stable anatomic reconstruction. Conceptional considerations support this idea, but more data are needed to identify outcome predictors.

## Introduction

### Hip arthroplasty and periprosthetic femoral fractures

Hip arthroplasty (HA) is a commonly performed successful procedure used to treat various hip pathologies. Due to the general aging of the population and increased lifestyle demands, the number of people receiving hip replacements is expected to further rise in the future. The United Nations’ 2017 report on World Population Prospects states “In Europe, 25% of the population is already aged 60 years or over and that proportion is projected to reach 35% in 2050, while in Northern America it will go from 22 to 28%” [1].

In general, complication rates for HA are low. However, periprosthetic femoral fractures (PFF) are a rare, but serious complication, that often occurs many years after the initial surgery. In the majority of patients, they are caused by minor trauma [2–5]. The risk to undergo revision surgery due to a PFF has been shown to increase with age, which becomes more evident in geriatric patients [6, 7]. Based on age being a major risk factor for osteoporosis and osteoporosis being a major risk factor for sustaining hip fractures [8], it is not surprising that the incidence of periprosthetic fractures around hip implants has steadily increased over the past few decades. The National Joint Registry of England and Wales reported a 1.5- and 1.6-fold increase in periprosthetic fractures around hybrid and cemented hip prosthesis respectively between 2011 [9] and 2016 [10] alone, while it remained stable for uncemented HAs.

Given the continual increase in volume of hip replacements performed and the lag between primary hip surgery and fracture, the number of periprosthetic fractures is expected to increase further in the years to come. The economic impact that arises from PFF is significant: Figures from the United Kingdom generated between 1999 and 2009 suggest that the average cost generated by a PFF amounts to £23,469 per patient [11].

### Treatment of PFF

Treatment of PFF is usually demanding, complication rates are high, and often the prognosis concerning outcomes and mortality is bad [5, 12]. The choice of the treatment modality depends on fracture characteristics, implant stability, the quality of the surrounding bone, and the presence of infection [13–15].

The Vancouver classification is currently the most commonly used classification for PFF after HA and provides distinct treatment recommendations. It divides PFF into three classes: A, B and C. Type A fractures are confined to the greater (A_G_) or lesser (A_L_) trochanter. Type B fractures are diaphyseal, around the prosthesis or immediately distal to it, and are divided into three subtypes: B1, B2 and B3, characterized by a well-fixed stem, an unstable or loose stem with good quality of the surrounding bone stock, and an unstable or loose stem with inadequate surrounding bone stock, respectively [13]. Type C fractures are well below the tip of the prosthesis.

According to the authors of the Vancouver classification, “type A fractures may be treated either conservatively or surgically, depending on the stability of the fracture. Type B1 fractures are best treated using ORIF or the bicortical onlay allograft technique. Type B2 fractures are best treated using long-stem revision total HA. Type B3 fractures usually require complex reconstruction. Type C fractures are best treated using ORIF” [13].

### Current treatment recommendations under debate

Recently, a debate around the necessity to strictly follow those recommendations for some fractures types has been raised. Several authors have argued that under certain conditions, B2 or B3 fractures could also be treated successfully with ORIF or ORIF combined with cement-in-cement revision [16–18].

Quah et al. have proposed a treatment algorithm where, in the presence of good bone stock, the potential to reduce the fracture anatomically as well as the integrity of the cement mantle rather than the stability of the prosthesis alone, are the major determinants for the treatment decision [18]. According to this algorithm, ORIF is an option for B2 fractures that can be reduced anatomically. Additionally, they point out that in cases where the cement is well fixed at the cement–bone interface, cement-in-cement revision could also reduce surgical time and better preserve the existing bone stock [17, 18].

The conception that ORIF could be advantageous in PFF around certain loose stems is supported by several reports where ORIF was compared to stem revision in B2 and/or B3 fractures and resulted in equivalent, if not better, outcomes [5, 16, 19–27].

To date, there is no publication that summarizes the factors determining the outcome after ORIF or revision arthroplasty in the treatment of B2 or B3 fractures based on clinical evidence. Therefore, we performed a systematic literature search with the aim to identify articles that provide sufficient information on potential prognostic factors for favorable or unfavorable outcome in the treatment of Vancouver B2 and B3 fractures.

## Materials and methods

### Search strategy

Systematic literature searches were conducted in Web of Science (WoS), Medline and the Cochrane databases in April 2017 and updated in February 2018 (Table 1).

**Table 1:** Search strategies in different medical databases

Database	Search strings	Matches
PubMed	(((periprosthetic) OR (*prosthetic) OR (interprosthetic)) AND ((vancouver b2) OR (vancouver b3) OR (vancouver)) NOT ((rat) OR (dog) OR (sheep) OR (rabbit) OR (pig) OR (experimental) OR (model)))	428
Web of Science	TS=(((periprosthetic) OR (*prosthetic) OR (interprosthetic)) AND ((vancouver b2) OR (vancouver b3) OR (vancouver)) NOT ((rat) OR (dog) OR (sheep) OR (rabbit) OR (pig) OR (experimental) OR (model)))	290
Cochrane libraries	Periprosthetic	277

### Study selection

Publications were deemed eligible if they were original research articles that included both patients treated with ORIF and patients treated with stem revision after a Vancouver B2 or B3 fractures and were published 1999 or later. In the case of mixed study populations (including Vancouver classifications other than B2 and B3 fractures), information on B2/B3 fractures had to be reported separately for both treatment groups to allow data extraction. Abstracts were only included if they presented sufficient numerical information to extract relevant data.

Additionally, the references of relevant review articles [12, 14, 18, 28, 29] were screened for further eligible studies.

### Data extraction and analysis

Information was collected on a standardized data extraction form and included the initial diagnosis for hip replacement, fixation parameters of the originally implanted stem (primary/revision, uncemented/cemented incl. fixation mechanism, e.g., cemented collarless polished tapered [CCPT]/composite beam [CB]), information on bone quality and stem stability at the time of PFF, and outcome parameters (e.g., union rate, functional outcome, complications, re-operations, mortality). Bearing in mind that our research question concerned explicitly B2 and B3 type fractures, relevant data were extracted specifically for B2 and B3 fractures, but not for other types of PFF.

### Data analysis

Depending on the type of information presented in the eligible publications, outcome data were collated stratified for treatment, i.e., ORIF and revision arthroplasty, as well as for the fracture types B2 and B3 separately. If needed, data from the publications were recalculated to facilitate presenting the desired stratifications. However, since the data presented in the eligible publications were scarce concerning prognostic factors and diverse concerning outcomes, it was not possible to quantitatively analyze summary data beyond the descriptive information in Table 2.

**Table 2:** Potential predictors and outcomes in the treatment of B2 and B3 periprosthetic fractures with osteosynthesis or a revision stem

Pub year Authors	Arthroplasty type, bone quality/Vancouver classification	Initial diagnosis	Fixation type Cemented: Composite Beam (CB) or polished taper (CCPT) Uncemented: proximal or distal fixation Both: primary or revision implant	Number of patients per treatment group (whole group, B2 and B3 fractures), implant details	Outcomes: hospital and surgery parameters	Outcomes: union rate and radiological outcome	Outcomes: functional outcome, mobility, disability	Complications and reoperations (for B2B3)	Survival	In favor of
2006 Lindahl et al.	**Vancouver classification:** A: 8 B1: 90 B2: 158 B3:34 C: 31 Cemented: 318 Uncemented: 3 **Primary THA:** Stable: 78 Loose^a^: 44 Unknown loose^b^: 108 **Revision THA:** Stable: 45 Loose^a^: 21 Unknown Loose^b^: 25	Not available	76% of the cohort used CB and CCPT stems	*Implant information for complete cohort:* **ORIF:** Unknown **Revision:** Cemented long stem: 144 Uncemented long, distal prosthesis: 49 *B2 and B3:* B2 Revision: 49 Revision + ORIF: 86 ORIF: 19 Other: 4 B3 Revision: 23 Revision + ORIF: 10 ORIF: 0 Other: 1	Not available	Not available	Outcomes (Harris hip score, pain VAS, survival) not provided separately for the treatment modalities or for B2 and B3 fractures (only for the complete cohort regardless of treatment group)	Complications not provided separately for treatment groups *Reoperations in B2* ORIF: 6/19 (32%) Revision + ORIF: 20/86 (23%) Revision: 5/49 (10%) *Reoperations in B3* ORIF: 0/0 Revision + ORIF: 2/10 (20%) Revision: 3/23 (13%)	Survival rates not provided separately for treatment groups	Re-operation rate for B2 in favor of revision (no ORIF patients for B3)
2010 Zuurmond et al.	A: 3 B1: 14 B2: 26 B3:7 C: 21	**Total: 71 patients** Primary osteoarthritis (71%) Fracture (15%) Avascular necrosis of the femoral head (6%) Rheumatoid arthritis (4%) Secondary osteoarthritis (4%) **B2: 26 patients** Primary osteoarthritis (77%) Fracture (15%) Avascular necrosis of the femoral head (4%) Rheumatoid arthritis (4%) **B3: 7 patients** Primary osteoarthritis (70%) Fracture (29%)	*Implants for B2 and B3:* **Osteosynthesis (incl. nail):** Initial primary THA in B2: 3 (2 CB, 1 uncem prox) Initial Primary THA in B3: 0 Initial Revision in B2: 3 (3 CB) Initial Revision in B3: 1 (1 CB) **Revision** Initial Primary THA in B2: 7 (4 CB, 1 uncem prox) Initial Primary THA in B3: 2 (2 CB) Initial revision in B2: 4 (4 CB) Initial revision in B3: 1 (1 CB) **Osteosynthesis and Revision** Initial Primary THA in B2: 9 (5 CB, 1 CCPT, 1 uncem distal, 1 uncem mainly proximal) Initial Primary THA in B3: 2 (1 CB, 1 CCPT) Initial Revision in B2: 0 Initial Revision in B3: 0	**Total group:** ORIF: 36 Revision: 14 Revision and ORIF: 14 Other: 7 **B2 and B3 fractures:** No information	Not available	*Fracture union rate:* No information available acc. to treatment type	*Mean Oxford hip score* (*n* patients with B2 or B3 and information on score): **Osteosynthesis** Initial Primary THA in B2 (*n* = 2): 17.5 Initial Revision in B2 (*n* = 2): 30.0 Initial Revision in B3 (*n* = 0) **Revision** Initial Primary THA in B2 (*n* = 5): 29 Initial Primary THA in B3 (*n* = 1): 39 Initial Revision in B2 (*n* = 2): 25.5 Initial Revision in B3 (*n* = 0) **Osteosynthesis and Revision** Initial Primary THA in B2 (*n* = 5): 27.2 Initial Primary THA in B3 (*n* = 1): 25	*Complications in patients with B2 or B3:* **Osteosynthesis** Initial Primary THA in B2: 2/3 (1 minor systemic, 1 major systemic) Initial Revision in B2: 2/3 (1 minor systemic, 1 femoral) Initial Revision in B3: 1/1 (1 femoral) **Revision** Initial Primary THA in B2: 4/7 (2 minor systemic, 1 major systemic, 1 femoral) Initial Primary THA in B3: 0/2 Initial Revision in B2: 2/4 (1 minor systemic, 1 major systemic) Initial Revision in B3: 0/1 **Osteosynthesis and Revision** Initial Primary THA in B2: 4/9 (2 major systemic, 2 femoral) Initial Primary THA in B3: 2/2 (2 minor systemic)	*2-year survival:* **Osteosynthesis** Initial Primary THA in B2: 2/3 Initial Revision in B2: 3/3 Initial Revision in B3: 1/1 **Revision** Initial Primary THA in B2: 6/7 Initial Primary THA in B3: 2/2 Initial Revision in B2: 3/4 Initial Revision in B3: 1/1 **Osteosynthesis and Revision** Initial Primary THA in B2: 2/9 Initial Primary THA in B3: 1/2	No information in favor of osteosynthesis or revision in B2 or B3 fractures available
2011 Pavlou et al.	A: 4 B1: 15 B2: 66 B3: 106 C: 11 All THA	Not available	No further information, e.g. primary or revision, CB or CCPT available	**Total group:** Conservative: 4 ORIF: 40 Stem revision: 158 **B2 and B3 fractures:** *ORIF:* B2 with grafting: 4 B2 without grafting: 10 B3 with grafting: 1 B3 without grafting: 6 *Stem revision:* B2 with grafting: 27 B2 without grafting: 25 B3 with grafting: 89 B3 without grafting: 10 No information on implants available	No further outcomes stratified for treatment (ORIF/revision) available	*Non-union/union* **ORIF** B2 grafting: 1/3 B2 no grafting: 4/6 B3 grafting: 0/1 B3 no grafting: 1/5 **Stem revision** B2 grafting: 1/26 B2 no grafting: 2/23 B3 grafting: 8/81 B3 no grafting: 1/9 ORs for all treatment combinations within a PFF type were calculated, only 2 were significant: B2 ORIF no graft vs. B2 stem revision and graft: OR 17.3; 95% CI 1.63,184.4; *p* = 0.018 B2 ORIF no graft vs. B2 stem revision no graft: OR 7.67; 95%CI 1.2,52.32; *p* = 0.038	No further outcomes stratified for treatment (ORIF/revision) available	*Infection/dislocation* **ORIF** B2 with grafting (4): 1/3 25%/75% B2 without grafting (10): 0/0 B3 with grafting (1): 1/0 100% B3 without grafting (6): 0/1 16.7% **Stem revision** B2 with grafting (27): 0/0 B2 without grafting (25): 2/3: 8%/12% B3 with grafting (89): 5/0 5.6% B3 without grafting (10): 2/0 20% *Reoperations:* No information provided	Not available	Union rate and complications (infections and dislocations): small n.s. trend in favor of revision, however patient numbers are very small
2013 Montalti et al.	Al: 3 PFFx Ag: 2 B1: 10 B2: 13 B3: 16 C: 3 **PFFx after primary THA: 36 patients** Cemented THA: 2 Cementless THA: 25 Hybrid THA: 9 **PFFx after previous revision: 11 patients** Cemented THA: 5 Cementless THA: 5 Hybrid THA: 3 (Note: unclear why the total isn’t 11.)	Osteoarthritis: 31 Femoral neck fracture: 6 Osteoarthritis secondary to developmental hip dysplasia: 4 Post-traumatic osteoarthritis: 4 Femoral head osteonecrosis: 2	No information on fixation mechanism available	**Total group:** ORIF: 11 Stem revision: 29 Conservative: 7 (Note: 10 patients had both stem and cup revision due to aseptic loosening) **ORIF** Plate/screw plus cerclage wires: 9 Cerclage wires only: 2 **Stem** Wagner SL Revision^®^ Hip Stem (Zimmer): 21 Restoration^®^ Modular Revision Hip System MT3 (Stryker): 5 Proformur^®^ Plasma Z Hip Stem (Wright): 3	Not available	Not available	Not available	**B3** Dislocation: 1 (?) Dtem breakage: 1 (?) Aseptic loosening: 1 PFF: 1 **B2** Dislocation: 1 Stem breakage: 0 Aseptic loosening: 1 PFF: 1 **B1** Aseptic loosening: 1 Intra-op PFF: 1 **C** Aseptic loosening: 1 PFF: 1	Not available	No information in favor of ORIF or revision in B2 or B3 fractures available since information on treatment group allocation is missing and information given on complications is discrepant
2014 Niikura et al.	Ag: 1 B1: 6 B2: 6 (1 of these treated conservatively) C: 5 THA or HA, not known for which pts Not known whether first HA or already revision	Not available	No information on fixation mechanism available	**Total group:** ORIF: 13 Stem revision: 2 Conservative: 3 **B2 and B3 fractures:** B2 fractures in 6 patients: *ORIF:* 3 Narrow LCP: 2 Reversed LCP-DF: 1 *Stem revision: 2* Cementless longer stem: 1 Cemented stem: 1 *Conservative treatment: 1*	*Surgery time for B2* **ORIF:** mean 152.6 min 205 min: 1 71 min: 1 182 min: 1 **Stem revision:** mean 144 min 138 min: 1 150 min: 1 *Intraoperative bleeding* **ORIF**: mean 390 g 615 g: 1 (4 units blood transf) 150 g: 1 405 g: 1 **Stem revision:** mean: 1502.5 g 535 g: 1 (2 units autol. blood tranf) 2470 g: 1 (12 units blood transf)	Bony union in all patients	*Parker Mobility Score for B2 only* **ORIF** 1 pt: Before fx 5, latest fu 5 1 pt: Before fx 1, latest fu 1 1 pt: Before fx 0, latest fu 0 **Stem revision** 1 pt: Before fx 9, latest fu 9 1 pt: Before fx 3, latest fu 3 *Ambulatory status* **ORIF** 1 pt: Before fx crutch, latest fu crutch 2 pts: Before fx non-ambulatory, latest fu non-ambulatory **Stem revision** 1 pts: Before fx no aids, latest fu no aids 1 pt: Before fx walker, latest fu walker *Social status* **ORIF** 1 pt: Before fx indep, latest fu indep 1 pt: Before fx with support, latest fu with support 1 pt: Before fx nursing home, latest fu nursing home **Stem revision** 2 pts: Before fx indep, lates fu indep	No infection (no further information given)	All patients with B2 and B3 survived *(2 cases of “early postoperative mortality 3 m and 5 m post-op, both type C)*	No reliable information in favor of ORIF or revision in B2 or B3 fractures available since sample too small N.s. trend that blood loss was less in ORIF
2014 Spina et al.	Ag: 1 B1: 30 B2: 7 B3: 11 C: 12 B2: Straight stem: 4 (+ 1 excluded) B2: Anatomic stem: 2 Not known for B3 Not known whether first HA or already revision	Not available	No information on fixation mechanism available	**Complete group:** *ORIF:* 54 *Revision: *7 **B2 and B3 fractures:** *ORIF:* 10 (+ 2 not included because of short FU): B2: 7 (+ 1) B3: 3 (+ 1) *Revision:* B3: 7	Not available	*Fracture consolidation and implant stability at the end of treatment* **ORIF** B2/straight stem/plate: 4 B2/anatomic stem/plate: 1 B2/anatomic/cables: 0 B3/NA/plate: 0 **Stem revision** B3: 7	*Functional outcomes* **ORIF** Walker/2 crutches/1 stick/walking free B2/straight stem/plate: 0/1/1/3 B2/anatomic stem/plate: 0/1/0/1 B2/anatomic/cables: 0/0/0/0 B3/NA/plate: 2/0/0/0 **Stem revision** B3: 1/2/0/4 *Mobility:* **ORIF** 4 blocks/stairs/indoors only B2/straight stem/plate: 3/4/5 B2/anatomic stem/plate: 1/2/2 B2/anatomic/cables: 0/0/0 B3/NA/plate: 0/0/2 **Stem revision** B3: 4/4/7 *Number of disabled pts* **ORIF** B2/straight stem/plate: 0 B2/anatomic stem/plate: 0 B2/anatomic/cables: 1 B3/NA/plate: 2 **Stem revision** B3: 0	*Complications (pts)* **ORIF** B2/straight stem/plate: 1 (1) B2/anatomic stem/plate: 3 (2) B2/anatomic/cables: 0 (0) B3/NA/plate: 0 (0) **Stem revision** B3: 3 (2) *Reoperations (pts)* **ORIF** B2/straight stem/plate: 0 (0) B2/anatomic stem/plate: 3 (2) B2/anatomic/cables: 0 (0) B3/NA/plate: 0 (0) **Stem revision** B3: 2 (2)	Information only available for the complete cohort: 1-year survival: 96.7% 2-year survival: 88.5%	No information in favor of ORIF or revision in B2 or B3 fractures available since treatment groups are too small (B3) or no comparison group (B2) is available
2015 Inngul and Enocson	B1: 23 B2: 25 B3: 1 C: 14 Number of HA/THA not provided for B2 and B3 PPF	Non-pathological femoral neck fracture Number of primary/revision not provided for PPF	CCPT only	**Complete group:** *ORIF: 44* Single lateral plate with screws: 25 Screws in combination with cerclage wires: 19 *Revision: 19* Longer cemented stem: 10 Longer cemented stem and lateral plate: 2 Distally fixed uncemented revision stem: 7 *B2:* ORIF: 9 Revision: 16 *B3:* ORIF: 0 Revision: 1	No outcome for B2 B3 provided	Not available	Not available	*Complications other than those re-operated upon not provided* *Reoperations in B2:* **ORIF:** 2/9 (1 re-fracture, 1 non-union) **Revision: **1/16 (1 re-fracture) *Reoperations in B3:* 0/1	Survival not provided separately for B2 and B3	No reliable information in favor of ORIF or revision in B2 or B3 fractures available due to missing information and small sample. Possibly a trend for less reoperations in stem revision
2015 Lunebourg et al.	B1: 18 B2: 23 B3: 2 Primary THA or HA, not known for which pts	Not available	No information on fixation mechanism available	**Complete group:** *ORIF: *35 Stainless steel anatomically curved plate (Aesculap, Tuttingen, Germany), 12 holes or 15 holes, cerclage wires, bicortical and monocortical srews *Revision stem and ORIF:* 8 Cemented stem (Arcad longue™, Symbios, Yverdon, Switzerland) High-viscosity cement (Palacos, Heraeus Holding GmbH, Hanau, Germany) **B2:** *ORIF:* 16 *Revision stem and ORIF:* 7 **B3:** *ORIF:* 1 *Revision stem and ORIF:* 1	*Surgery time* **ORIF** B2 (*n* = 16): mean (SD): 122 min (26); range 80–165 B3 (*n* = 1): 90 min **ORIF and stem revision** B2 (*n* = 7): mean (SD): 209 min (41); range 165–278 B3 (*n* = 1): 185 min *Blood loss* **ORIF** B2 (*n* = 16): mean (SD): 456 mL (159); range 200–650 B3 (*n* = 1): 250 mL **ORIF and stem revision** B2 (*n* = 7): mean (SD): 667 mL (337); range 200–1000 B3 (*n* = 1): 750 mL	Union achieved in all patients	No further outcomes acc. to treatment group available	*Complications:* none mentioned *Reoperations in B2 and B3* **ORIF** 1 due to aseptic loosening of the femoral stem associated with acetabular protrusion 3 years later (in a B2 pt with HA) treated with ORIF alone **unknown group:** 1 due to deep infection after 4.5 years (in a B2 pt with HA)	Not available	Surgery time and blood loss seemingly in favor of ORIF, however patient numbers are small
2015 Solomon et al.	**B2: 21** ORIF: 12 (all primary THA) Stem revision: 9 (7 primary, 2 revision THA)	Not available	**Only CCPT stems:** **ORIF (all primary THA)** CPT stems (Zimmer Ltd, Warsaw, IN, USA): 6 Exeter stems (Stryker Howmedica Osteonics, Berkshire, UK): 6 **Stem revision (7 primary, 2 revision THA)** CPT stems (Zimmer Ltd, Warsaw, IN, USA): 4 Exeter stems (Stryker Howmedica Osteonics, Berkshire, UK): 5	*ORIF: 12* Cable ready plates, cables/cerclage, non-locking screws ((Zimmer Pty Ltd, Warsaw, IN, USA): 11 Cables/cerclage: 1 *Stem revision: 9* Long cemented stem with cable fixation: 4 Long cementless stem with distal fixation, cables for prox femur: 5	*Operating room time (p* = *0.002)* **ORIF** Median (range): 183 min (143–239) **Stem revision** Median (range): 270 min (206–352) *Skin-to-skin surgical time (p* *= 0.002)* **ORIF** Median (range): 122 min (80–165) **Stem revision** Median (range): 200 min (142–285) *No (range) of transfusion units (p* = *0.008)* **ORIF:** 0 (0–4) **Stem revision: **3 (0–5)	Anatomical reduction achieved in all cases *Radiographic follow-up* **ORIF (9 pts)** All fx healed Total stem subsidence at latest follow-up: Manual measurement: < 3 mm Ein-Bild-Roentgen-Analyse (EBRA): < 6 mm No stem loosening, no femoral osteolysis **Stem revision** No complications on radiographs	*Functional FU, Harris pain score *(subscore of the Harris Hip Score) has 44 as maximum (= none or ignores it) **ORIF** Median (range): 44 (20–44) **Stem revision** Median (range): 40 (10–44) *Mobility* **ORIF** Return to pre-injury levels around the house, out of the house, when shopping: 3 pts Pts unable to return to pre-injury levels required a walking aid to mobilize **Stem revision** Return to pre-injury levels around the house, out of the house, when shopping: 5 pts	*Complications:* **ORIF** **(*n*** = **12)** Episode of 2 dislocations, 4 yrs post-op: 1 pt **Stem revision (*n*** = **9)** Delayed wound healing: 2 pts Episode of 2 dislocations each, within the first 3 post-op months: 2 pts *Reoperations:* None mentioned	**ORIF: **9/12 **Stem revision: **7/9 All 5 deaths occurred within the first 3 months, before fx healing 3 pts had an ASA score of 4 and 2 pts had an ASA score of 3 All causes were unrelated to hip surgery	Significantly in favor of ORIF for OR time, skin-to-skin time, blood transfusions N.s. trend in favor of ORIF for Harris pain score and for mobility in favor of revision (but mean pt age 9 years less and 1 pt evaluated directly after knee replacement) N.s. trend for complications in favor of ORIF However patient numbers are small
2016 Joestl et al.	B2: 36 (Revision THA: 2 Primary THA: 34)	Not available	No information on fixation mechanism available	*ORIF: 8* 4.5 mm LCP *Stem revision (uncemented): 28* Hyperion (second generation) stem: 14 Helios (first generation) stem: 14 Helios^®^: Biomet, Europe, Valencia, Spain Hyperion™: Biomet, Europe, Valencia, Spain	*Surgery time (p* = *0.1025)* **ORIF** Mean (range): 151 min (90–205) **Helios revision** Mean (range): 190 min (135–355) **Hyperion revision** Mean (range): 191 min (130–260) *Blood units needed (p* = *0.0754)* **ORIF: **5 **Helios revision: **12 **Hyperion revision: **6 *Overall length of hospital stay (p* = *0.4748)* **ORIF: **mean (range): 26 days (11–49) **Helios revision:** mean (range): 26 days (11–55) **Hyperion revision: **mean (range): 29 (19–70)	Not available	Parker Mobility Score (*p* = 0.2940) **ORIF** Mean (SD): 6.62 (2), range 4–9 5/8 pts were back to their pre-injury levels of mobility around the house, out of the house and when shopping **Helios revision** Mean (SD): 6.5 (2), range 4–9 8/14 were back to pre-injury levels in all 3 categories **Hyperion revision** Mean (SD): 6.35 (2), range 4–9 7/14 were back to pre-injury levels in all 3 categories	*Complications* **ORIF** No complications **Helios revision** 1 dislocation of prothesis during a fall (closed reduction) 1 surgical- side infection and fistula due to a fall (soft-tissue revision). **Hyperion revision** 2 spontaneous dislocations (closed reduction) 1 superficial periprosthetic joint infection with Staphylococcus aureus (surgically revised) *Reoperations* **ORIF** No reoperations **Helios revision** 1 soft-tissue revision due to a surgical- side infection and fistula due to a fall **Hyperion revision** 1 surgical revision of a superficial periprosthetic joint infection with Staphylococcus aureus	all patients	N.s. trends in favor of ORIF for surgery time, blood units needed and number of patients returning to pre-injury activity level, however patient numbers are small
2017 Antoniadis et al.	B2	Not available	No information on fixation mechanism available	Revision: 26 ORIF: 27	*Surgical time *(*p* = 0.0187) **Osteosynthesis**: 124.5 min (range 75–194) **Revision**: 150 min (range 96–241) *Blood loss* (*p* = 0.0422) **Osteosynthesis**: 500 ml (range 50–1500) **Revision**: 700 ml (range 250–5200) *Need for further surgery* **Osteosynthesis**: 2	Not available	Not available	Odds ratio for having a complication, revision group: 2.449 (95% CI: 0.7654–7.668)	Odds ratio of dying within first 12 months was 6.19 (95% CI 0.6705–57.15) after stem revision	Surgical time and blood loss significantly in favor of ORIF N.s. trend in favor of ORIF for complications and mortality
2017 Baum et al.	B2: 57 (THA: 53 HHA: 4)	Not available	No information on fixation mechanism available	Revision: 36 ORIF (LCP): 21	*Length of hospital stay* **ORIF** (*n* = 21): 17 d **Revision** (*n* = 36): 19 d *Blood transfusio*n **ORIF** (*n* = 21): 16 pts **Revision** (*n* = 36): 27 pts *Average OP time* **ORIF** (*n* = 21): 146.7 min (95–205) **Revision** (*n* = 36): 152.6 min (70–255)	*Non-union* **ORIF** (*n* = 21): 1 **Revision** (*n* = 36): 8	Not available	*General complications:* **ORIF** (*n* = 21): 6 **Revision** (*n* = 36): 9 *Implant-related complications:* **ORIF** (*n* = 21): 2 **Revision** (*n* = 36): 8 *Revision surgery:* **ORIF** (*n* = 21): 5 **Revision** (*n* = 36): 9	Not available	Trends in favor of ORIF for union rate, implant-related complications, blood transfusion and possibly surgery time and length of hospital stay
2017 Bulatovic et al.	**B1: 10** cemented: 8 uncemented: 2 **B2: 10** cemented: 7 uncemented: 3 **B3: 3** cemented: 3	Not available	No information on fixation mechanism available	**B1** LCP: 6 DCP: 3 wire cerclage: 1 (Complication: 4 patients) **B2** LCP: 5 DCP: 2 Long stem: 1 cerclage: 2 **B3** LCP: 2 Long stem: 1	Not available	Not available	Modified Merle d’Aubigné score B2: 10.6 (bad) B3: 12.0 (fair)	**B2 LCP** Broken plate/re-op: 1 Pressure ulcers on skin: 1 Broken LCP/re-op: 1 Deep vein thrombosis: 1 **B2 DCP** Broken plate/re-op: 1 Superficial wound infection: 1 **B2 cerclage** Wound infection and ulcer on the skin: 1 Death: 1 (after 1 yr) **B3 LCP** Broken plate/re-op: 1 (at 3 mo) Superficial wound infection: 1	22 patients at 1 yr	No reliable information in favor of ORIF or revision in B2 or B3 fractures available due to missing information and small sample (revision only in 1 patient each in B2 and B3)
2017 Gitajn et al.	B1: 82 B2: 96 B3: 25	Not available	No information on fixation mechanism available	**Complete group:** ORIF: 110 Revision arthroplasty: 93 **B2 and B3 combined:** ORIF: 42 Revision: 79 Implants not known	*B2 and B3 combined* **ORIF:** estimated blood loss (EBL): 585 cc (439) No. blood units transfused: 2.8 (2.2) **Revision THA:** EBL: 1168 cc (569) No. blood units transfused: 5.0 (3.5) both parameters: *p* < 0.001	Not available	*Weight bearing instructions (B2 and B3 combined):* **ORIF**: NWB (no): 2 TDWB (touch down/toe touch): 24 PWB (partial): 9 WBAT (as tolerated): 7 **Revision THA:** NWB: 1 TDWB: 14 PWB: 16 WBAT: 48	No information provided for reoperations No information provided for complications of B2B3	*B2 and B3 combined:* **Survival **in patients treated with surgical fixation compared to revision arthroplasty: No difference at 1 year (83% vs. 85%, *p* = 0.76) or 5 years (41% vs. 58%, *p* = 0.11)	Blood loss and blood transfusions in favor of ORIF, Postoperative weight bearing instructions in favor of revision No difference in survival

^a^Known as loose beforehand, scheduled for revision

^b^Recognition of loose at presentation

## Results

Our systematic search identified 14 original studies that included patients treated with ORIF and patients treated with revision arthroplasty in the treatment of B2 and B3 PFF [5, 16, 19–27, 30–32]. The publications were mainly retrospective cohort studies which did not describe the treatment allocation, provided little information about potential predictive factors and presented diverse outcome parameters. Additionally, in most studies, the group sizes were small so that no statistical tests were performed. Table 2 provides an overview about the eligible studies including relevant outcomes and potential predictors.

### Prognostic factors

Only a handful of publications provided information about the most likely prognostic factors such as type of the original stem, initial indication for THA, or implants used to treat the PFF, which have been shown to be important outcome predictors [29]. Therefore, it was not possible to quantitatively analyze these potential prognostic factors.

Eight studies presented information on whether the PFF occurred around primary or around revision hip stems [5, 16, 22–25, 27, 31], eight presented the information whether the stem was cemented or uncemented [5, 16, 22, 24, 25, 27, 30, 31], three provided information about the fixation mechanism of the cemented stem (CCPT or CB) or the stem’s brand name for the respective treatment group [16, 22, 27], three informed about the diagnosis that had led to the initial hip replacement [22, 27, 31] and 7 provided details about the implants used for ORIF [16, 20, 22–25, 30]. One study presented some of the aforementioned parameters for some of the PFF types but did not follow a consistent pattern in doing so [26].

In addition to the assessment of bone quality and stem stability inherent to the Vancouver classification (B2: unstable or loose stem with good quality of the surrounding bone stock, B3: unstable or loose stem with inadequate surrounding bone stock), one study classified the bone quality around B3 fractures according to Paprosky [5]. In this study no ORIF was used to treat B3 fractures.

### Outcome parameters

Since the reported outcome measures were diverse, it was not possible to summarize them quantitatively. Some studies focused on mobility [16, 23–27, 30] as measured with various instruments, whereas other studies focused on union rates [16, 20, 23, 24, 26, 27, 32], reoperation rates [5, 19, 21–23, 30, 32], mortality [21, 22, 25, 26], or on perioperative parameters [16, 19–21, 23–25], including various combinations of these outcomes.

### Success of treatment modality

Only five studies used statistical tests to compare the results of ORIF vs. stem revision in B2 or B3 fractures. Solomon et al. compared ORIF to stem revision in 21 patients whose initial stem was a CCPT stem and who had sustained B2 fractures. The results were significantly in favor of ORIF for surgical time and the need for blood transfusions. There were also non-significant (n.s.) trends in favor of ORIF for the Harris pain score and complications as well as n.s. trends in favor of revision surgery for mobility. However, the authors pointed out that the mean age in the revision group was 9 years higher and that one of the nine patients in the revision group had been evaluated for mobility directly after having received a knee replacement to explain the latter trend [16]. Antoniadis et al. compared results of ORIF to stem revision in 53 patients who had sustained B2 fractures with an unknown fixation type of the initial stem. They found results significantly in favor of ORIF for surgical time and blood loss along with n.s. trends in favor of ORIF concerning complications and mortality [19]. Joestl et al. compared the results of 3 treatment modalities to treat B2 PFF, one being ORIF, with a group size of only 8, and two different revision stem designs with group sizes of 14 each. They found n.s. trends in favor of ORIF for surgery time, blood units needed and the number of patients returning back to their pre-injury mobility level [23]. Gitajn et al. analyzed the outcomes of patients with B1, B2 and B3 fractures. The results of the patients who had sustained B2 or B3 fractures were in favor of ORIF concerning blood loss and blood transfusions, showed no difference concerning mortality, and were in favor of revision surgery concerning the postoperative weight bearing instructions given by the treating practitioner [21]. Pavlou et al. compared ORIF with and without grafting with stem revision with and without grafting, amongst other PFF types also between B2 and B3 fractures. The main outcome of the paper was that in the treatment of PFF, using a graft in addition to ORIF or a revision stem is beneficial. A comparison of all possible treatment combinations (ORIF/revision/with graft/without graft) demonstrated that in B2 fractures, treatment with a revision stem with or without graft resulted in higher union rates than treatment with ORIF without graft. No significant differences were seen in any of the other combinations, including ORIF with graft compared to revision with a stem with or without graft [32].

In several other publications, which had not used statistical tests to compare the results of ORIF vs. stem revision in B2 or B3 fractures, there were trends which appeared to favor ORIF for various parameters [20, 24, 25]. Other publications also showed trends for better outcomes of revision arthroplasty [22, 24]. However, the lack of appropriate statistics and the small group sizes do not allow drawing reliable conclusions.

## Discussion

In summary, the majority of recent studies suggest equivalence of ORIF compared to revision arthroplasty in the treatment of selected B2 and B3 fractures. Notwithstanding, most of these studies have significant methodological weaknesses, especially concerning the choice of treatment allocation, which bears a high risk of producing biased results. Additionally, the scarcity of information concerning prognostic factors and the diversity of outcome instruments did not allow to identify outcome predictors or to quantitatively summarize the results.

This is well in line with the findings of Mont and Maar, who published a quantitative analysis of the outcomes after PFF reported in the literature in 1994 [33]. They found that the lack of relevant information provided in the respective publications rendered them unable to stratify the results for important predictors.

Only few studies provided a rigorous statistical analysis [16, 19, 21, 23, 32]. Whereas some of these studies could show significant advantages of ORIF for various in-hospital parameters [16, 19, 21], this was not the case for postoperative clinical outcomes: Only one study found a significant difference, namely the postoperative weight bearing instructions as given by the treating physician, which allowed more weight bearing after revision arthroplasty than after ORIF, and thus was in favor of revision surgery [21]. Various studies found n.s. trends in postoperative outcome parameters either in favor of ORIF [19, 23], in favor of revision [32] or balanced [16], i.e. some parameters were in favor of ORIF; others in favor of stem revision. These findings challenge the recommendations given in the Vancouver classification, which defines that B2 and B3 PFF are associated with an unstable prosthesis and thus should be replaced with a long-stemmed prosthesis [13].

In line with the findings of the aforementioned studies, several authors postulate that B2 or B3 fractures could also be treated successfully with ORIF [16, 18]. This would not need to be restricted to palliative care in patients with low lifestyle demands. A fixation of PFF with ORIF alone could be generally beneficial due to the reduced surgical time and complexity and reduced implant costs. Moreover, preserving bone stock by avoiding a long-stemmed implant could benefit especially younger patients who are likely to require further revisions in the future [16].

In B2 fractures, provided there was sufficient bone stock and the possibility to anatomically reconstruct the fracture, a construct with sufficient stability to allow fracture healing could be created by means of ORIF [16, 18]. In particular straight polished tapered stems could regain stability after anatomic fracture reduction within an intact cement mantle [16]. The principle of the “loaded taper” design of cemented stems is also referred to as “force-closed” fixation where the polished stem does not bond with the surrounding cement und during axial loading becomes lodged as a wedge in the cement mantle. These stems are polished and do not bond with the surrounding cement. Thus, they allow the taper to subside within the cement mantle until it stabilizes, even in case of anatomically reduced periprosthetic fractures. In primary THA, these stems usually stabilize after the first year [34–36]. From the literature it is unknown how long it takes the stem to stabilize once the fracture has healed in an anatomical way.

In the other kind of cemented stem, the “composite beam” type (also referred to as “shape-closed” fixation), a firm cement–metal bond is mandatory. These implants are not intended to subside, and may either be collared or uncollared, anatomical or straight, undersized or canal-filling. All these designs have in common that the strong bond between the interfaces of stem and cement as well as the interface of cement and bone creates a stable construct from three different materials with different elasticities. Because the stem possesses the highest stiffness, it transmits the axial load to its tip and to the cement and bone below it [37]. Once the interface between the stem and the cement is disrupted, a loose stem can by definition never become stable again. In uncemented stems, long term clinical success depends on stem stability, which in turn depends on the area of primary fixation. According to the Mont group, the uncemented stems can be categorized in 6 major design types, primarily defined by the location of fixation (metaphyseal, diaphyseal or a combination of both) as well as their design geometry [38]. If the periprosthetic fracture does not involve the primary location of fixation, the stem remains stable and the fracture can be internally fixed. However, if the primary area of the implant stabilizing zone is involved in the fracture, the stem is loose and it will depend on the type of fracture and implants used whether sufficient stabilization can be achieved or not. According to Pilliar et al., micromotion of < 20 µm results in predominantly bone formation, more than 150 µm leads to fibrous tissue formation and between 40 and 150 µm results a combination of bone and fibrous tissue formation [39]. In an unstable fracture fixation, the excessive micromotion will inhibit osseointegration of uncemented components and the stem will remain unstable, causing thigh pain associated with possibly reduced range of motion and a limp [40].

Considering the multitude of anchoring philosophies, understanding the underlying biomechanics of the fixation is essential to determine whether ORIF may be a suitable treatment. These technical considerations on stem fixation philosophies provide insights in potential success or failure mechanisms for PFF fixation.

The main limitation of our work lies in the low quality of publications. None of the eligible publications was clearly prospective and only few studies used statistical tests to identify differences in outcomes of B2 and B3 fractures treated with ORIF or stem revision. Moreover, not a single study applied transparent decision algorithms for treatment allocation that would have resulted in balanced groups, hence the risk of biased results is high. Beyond this, the lack of uniform outcome reporting did not allow to synthesize any outcome results and the lack of documentation of potential outcome predictors made it impossible to analyze those.

Notwithstanding, the currently available best evidence implicates there may be a benefit of ORIF vs. stem revision. However, the scarcity of information about prognostic factors makes it clear that further research to determine the relevant predictors is badly needed. Therefore, a registry that will document relevant predictors as well as outcomes after treatment of PFF along with other periprosthetic fractures is being funded by the AO Foundation via the AO TK Trauma and AOTrauma Network and set up by AOCID (registration: ClinicalTrials.gov: NCT03378557).

The documentation will include information about initial implant and pathology, fracture classification, clinical, radiological and intraoperative stem stability, patient data (comorbidities, joint function, pain, quality of life), adverse events, implants used to treat the PFF, quality of fracture reduction, fracture healing and postoperative clinical and prosthetic loosening. In the first phase a limited number of sites from different regions will participate. Once launched successfully, it is envisioned to open the registry for further sites.

## Conclusion

PFF are a rare but important complication of hip arthroplasty. The choice of the treatment modality depends on fracture, implant, and bone characteristics. Recent data challenge the present treatment recommendation as they show that successful outcomes can be achieved without revising loose stems. ORIF may be a viable option in fractures around uncemented stems as well as around polished tapered stem designs with an intact cement mantle if a stable anatomic reconstruction is deemed possible. Conceptional considerations form the basis of this idea, which is further supported by results from the recent literature. Notwithstanding, due to methodological weaknesses of the available studies, the rising evidence confirming the benefits of ORIF is still weak and more data are needed to identify relevant outcome predictors.
